# The Genome of the Rice Variety Mowanggu Provides Insight Into Resistance to *Magnaporthe oryzae*

**DOI:** 10.1111/mpp.70223

**Published:** 2026-03-26

**Authors:** Weiye Peng, Pingyong Sun, Nuan Yi, Zhuozhi Zheng, Bing Wang, Jing Liu, Xionglun Liu, Liangying Dai, Wei Li, Yunsheng Wang

**Affiliations:** ^1^ Hunan Provincial Key Laboratory for Biology and Control of Plant Diseases and Insect Pests Hunan Agricultural University Changsha Hunan China; ^2^ College of Plant Protection Hunan Agricultural University Changsha Hunan China

**Keywords:** evolution, genome assembly, *Magnaporthe oryzae*, plant immunity

## Abstract

Food security remains a pressing global challenge, particularly for staple crops like rice. The traditional Yunnan landrace rice variety Mowanggu (MWG) exhibits broad‐spectrum and durable resistance to *Magnaporthe oryzae*, the causal agent of destructive rice blast disease, making it a valuable germplasm resource for breeding. However, the molecular mechanisms underlying this resistance remain unclear due to the lack of a high‐quality genome. Here, we present a chromosome‐scale draft genome assembly of MWG, combining Nanopore long‐read and Illumina short‐read sequencing. Through comparative genomic analyses, we identified structural variations, gene family expansions and divergence events. We identified nine RLK genes within the *Pi49* resistance locus, among which overexpression of *OSAmwg_038136* enhanced the expression of pathogenesis‐related genes and increased resistance to rice blast. OSAmwg_038136 was shown to interact with OsDIP1, a member of the R3H protein family, which positively regulates blast resistance. Our findings provide critical insights into the molecular basis of MWG durable blast resistance and offer a foundation for engineering broad‐spectrum disease resistance in rice.

## Graphical abstract

Chromosome‐scale Mowanggu genome sequence reveals durable, broad‐spectrum rice blast resistance; expansion of RLK/NBS genes at *Pi49*, with OSAmwg_038136 enhancing defence and interacting with OsDIP1 to regulate resistance.

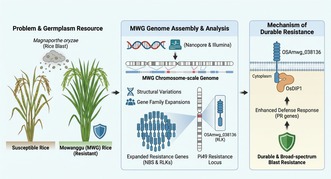

## 1 Introduction

Rice ( *Oryza sativa* ) is one of the most widely cultivated staple food crops in the world, consumed by nearly half of the global population (Hou et al. 2024). However, rice production is severely threatened by various diseases, among which rice blast, caused by the fungal pathogen *Magnaporthe oryzae*, is one of the most devastating (Jiang et al. 2024; Zhang et al. 2024). Rice blast can lead to significant yield losses, ranging from 10% to 30% annually, and in severe cases, complete crop failure (Guo et al. 2025). Rice was domesticated from its wild ancestor, *Oryza rufipogon* , a widely distributed native species of Asia, over 8000 years ago (Zheng et al. 2024). The genetic diversity of domesticated rice is significantly reduced in comparison to *O. rufipogon* (Wang, Chen, et al. 2024). The most significant loss of genetic diversity occurred in high‐yield semi‐dwarf modern rice varieties, which resulted in the lack of evolutionary adaptation to varying environmental conditions across lineages (Huang et al. 2024; Zhang et al. 2020). Compared to modern improved rice (IR) varieties, traditional landraces of rice (LR) preserved by local farmers can be considered as a transition phase of domestication between wild and cultivated rice, and they act as natural reservoirs of genetic variation (Long et al. 2024; Higgins et al. 2022). These LRs are especially important for the future improvement of germplasm because they can be employed to create novel varieties with improved adaptation capacity to biotic and abiotic stress (Han et al. 2020; Yang et al. 2022).

However, over the last few decades, local LRs have been increasingly replaced by genetically uniform IRs in most planting areas of China except for a small region in the southwest (Gladieux et al. 2024). Yunnan Province, located in southwestern China, is known for its high biodiversity and is home to many morphologically, functionally and genetically diverse LRs (Qian et al. 2020; Gladieux et al. 2024). Mowanggu (MWG) is a long‐cultivated *japonica* cultivar from Yunnan with broad‐spectrum and durable blast‐resistance (Peng et al. 2023). In addition, MWG exhibits favourable agronomic characteristics and displays tolerance to abiotic stress, rendering it a promising candidate for breeding purposes (Sun et al. 2022).

High‐quality reference genomes can provide massive genetic and genomic information for promoting biodiversity research, allowing plant breeders to conduct functional genomics, descriptive genomics and molecular breeding using diverse methods (Zhang et al. 2024). The genome of rice is smaller on average compared to that of other graminaceous crops, such as maize, barley and wheat (Du et al. 2017; Wei et al. 2024). Previous research has revealed significant genomic variation, particularly structural variations (SVs), among different *Oryza* species. These variations have become increasingly evident with continued advances in sequencing technologies and analytical algorithms (Kou et al. 2020; Song et al. 2024). The emergence of high‐quality genomes for 3010 Asian cultivated rice accessions has facilitated the identification and cloning of resistance (*R*) genes, along with small polymorphisms (Wang et al. 2018). Nonetheless, a comparative pan‐genomic analysis involving 66 cultivated and wild rice samples from different regions in China has led to the discovery of 23 million sequence variants, in addition to a considerable number of insertions/deletions (InDels) and variations in presence/absence (Zhao et al. 2018). In addition, third‐generation long‐read sequencing combined with second‐generation sequencing has been used to assemble the genomes of 251 Asian and African cultivated rice accessions. This effort has generated annotated genomes and comprehensive structural variation maps, thereby enhancing our understanding of rice molecular architecture and facilitating the utilisation of elite rice germplasm resources (Shang et al. 2022). Despite the abundance of reference genomes from modern cultivars, the genomic landscape of traditional landraces remains poorly characterised, creating a critical bottleneck in deciphering the genetic architecture of disease resistance.

In the present investigation, we established the first *de novo* genome assembly of the durably resistant rice cultivar MWG through a combination of Nanopore long‐read and Illumina short‐read sequencing technologies. This assembly offers a quality reference genome that can be used as a reference for future research on MWG and allow accurate analysis of MWG‐related omics data. We also conducted genome functional annotation, evolutionary analysis, comparative genomics and identified genes and pathways associated with rice blast resistance. The information contained in the MWG genome sequence and annotation may promote the sequencing and assembly of genomes of other rice varieties in the Rice Genomes Project, and it will also enhance numerous genetic studies based on the genetic background of MWG. Our research contributes to a better understanding of the regulatory mechanisms of the rice– *M. oryzae* pathosystem and reveals an important strategy for future genetic engineering to improve blast resistance.

## 2 Results

### 2.1 Genome Sequence and Assembly

**FIGURE 1:**
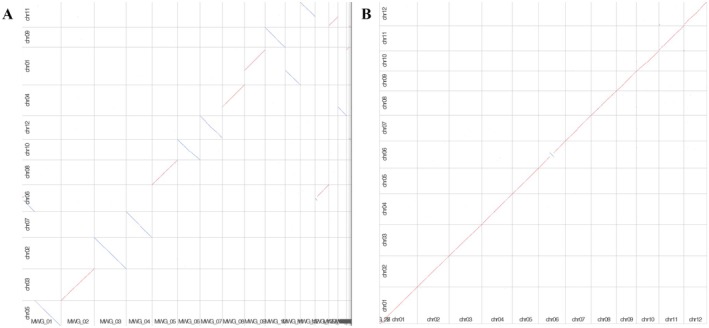
Overview of the rice variety Mowanggu (MWG) genome assembly. (A) Dot‐plot of MWG contigs against rice reference genome Nipponbare (NPB). The MWG contigs are arranged by size along the *x*‐axis and the NPB chromosomes are along the *y*‐axis. Forward‐strand matches are shown as diagonal lines from bottom left to top right and reverse‐strand matches are shown as diagonal lines from top left to bottom right. (B) Dot‐plot of MWG chromosomes against rice reference genome NPB. The MWG chromosomes are arranged by size along the *x*‐axis and the NPB chromosomes are along the *y*‐axis. Forward‐strand matches are shown as diagonal lines from bottom left to top right and reverse‐strand matches are shown as diagonal lines from top left to bottom right.

For accurate genome assembly, we used a combination of protocols and sequencing technologies, including long‐read Oxford Nanopore sequencing and short‐read Illumina sequencing platforms. The final assembled size of the MWG genome after genome primary assembly, polishing and de‐redundancy was 380.5 Mb in 27 contigs with an N50 of 25.67 Mb. The MWG assembly contigs were aligned to the Nipponbare (NPB) reference genome, and 7 (chr02, chr03, chr07, chr08, chr09, chr10, chr12) out of the 12 chromosomes were assembled into one contig each (Figure 1A). Chr01, chr04 and chr11 were assembled into two contigs each, chr01 (MWG_09, MWG_11), chr04 (MWG_08, MWG_15) and chr11 (MWG_12, MWG14). While the overall assembly exhibits high continuity, one chimeric contig (MWG_01) was identified, probably resulting from the merging of highly similar pericentromeric repeats shared between chromosomes 5 and 6, a known assembly complication. We assembled the MWG genome into 12 chromosomes using the NPB genome as a reference and the two genomes displayed strong lineal synteny (Figure 1B). BUSCO assessment revealed that approximately 98.2% of the core conserved Poales genes (poales_odb10) were in their complete forms in the MWG assembly. A total of 43,467 coding genes were annotated in the MWG genome, with an average exon number per gene of 3.95 and an average exon length of 251.37 bp, which is comparable to other released rice genomes (Table S2). The gene density, GC rates (average 43.54%) and SNP/indels (1,039,581) along each contig were calculated, and their distributions were uneven (Figure S1).

### 2.2 Whole‐Genome Comparisons of MWG to Other Rice Varieties

To characterise the segmental duplication and differences between MWG and NPB, we performed synteny and collinearity analysis between the MWG genome and the reference genome (Figure S2A). There were 901 duplication events in the genome of MWG, and contig 1, 3 showed high macrosynteny with NPB (Figure S2B). Comparative genomic analysis revealed high collinearity between MWG and the reference genome NPB, but low collinearity with the *indica* genome CO39 (Figure S3). In particular, we discovered small‐scale SVs between MWG and the *japonica* or *indica* genomes. The notable exception was an apparent inversion in the MWG genome assembly at chromosome 6 between 12.7 and 17.6 Mb (NPB coordinates) and 13.1 and 18.6 Mb (CO39 coordinates), which corresponds to the region the centromeric region.

### 2.3 Resistance‐Related (*R*) Genes

**FIGURE 2:**
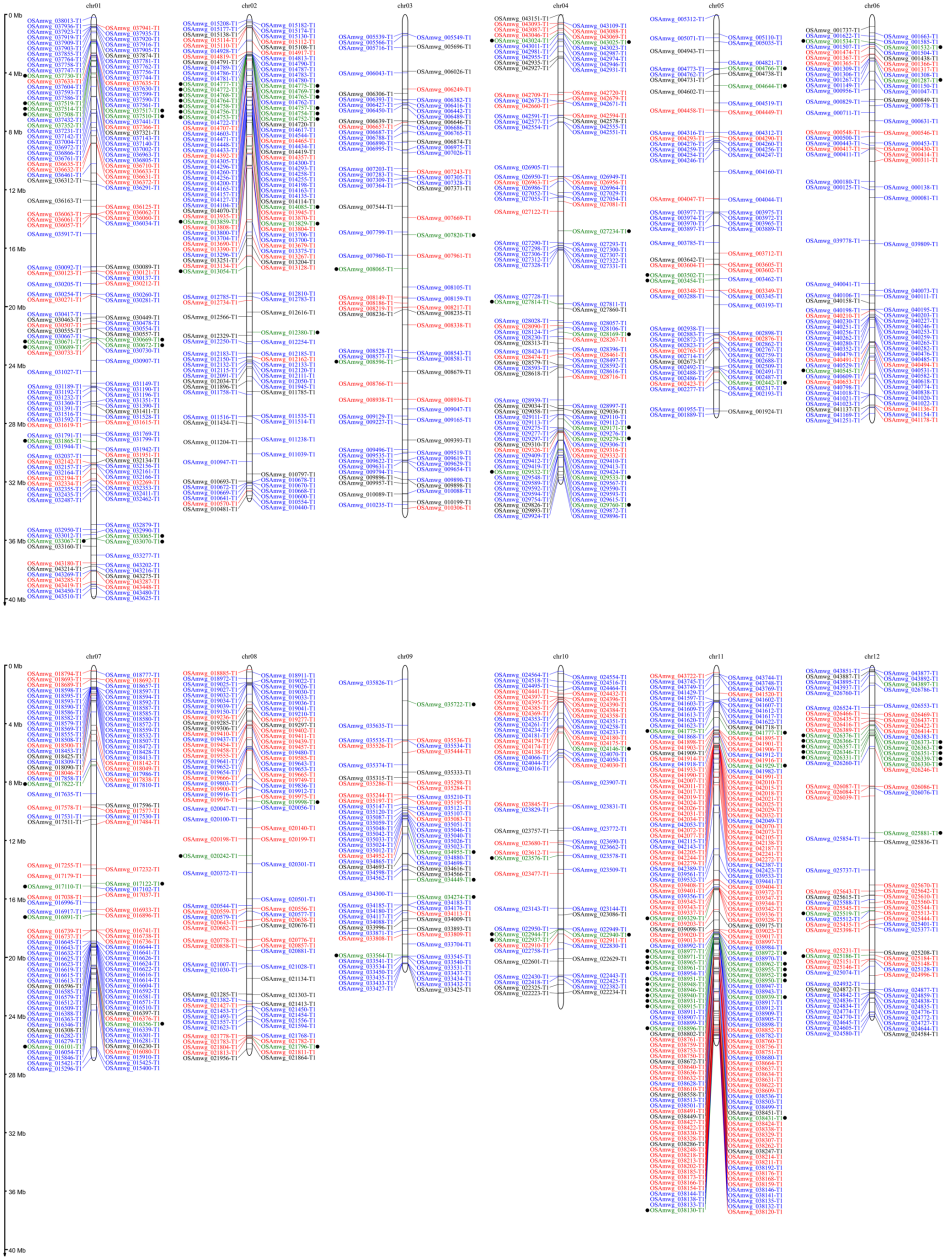
Identification of resistance‐related gene family in the rice variety Mowanggu (MWG) genome. The lines in red, blue, green (with black circles) and black represent the NBS, RLK, RLP and TM‐CC genes, respectively. The numbers at the top of the panel indicate rice chromosomes.

Plant *R* genes are crucial gene classes that generally consist of nucleotide‐binding site (NBS) and transmembrane‐coiled‐coil (TM‐CC), receptor‐like kinase (RLK) and receptor‐like protein (RLP) and play an essential role in plant disease resistance. In the MWG genome, 352 NBS (227 in NPB), 728 RLK (659 in NPB), 111 RLP and 128 TM‐CC with complete domains were detected (Figure 2). Among them, the NBS family is mainly distributed on chromosome 11, the RLP family is mainly distributed on chromosomes 2, 11 and 12, the RLK family is mostly distributed at both ends of the chromosome, and the TM‐CC family is randomly distributed across all chromosomes. In addition, we performed phylogenetic analysis of MWG RLK and NBS family genes. Notably, some NBS and RLK form a separate evolutionary branch, like *Pi49* loci, suggesting distinctive functions (Figure S4).

### 2.4 Comparative Genome Analysis of *Pi49* Localization Regions

**FIGURE 3:**
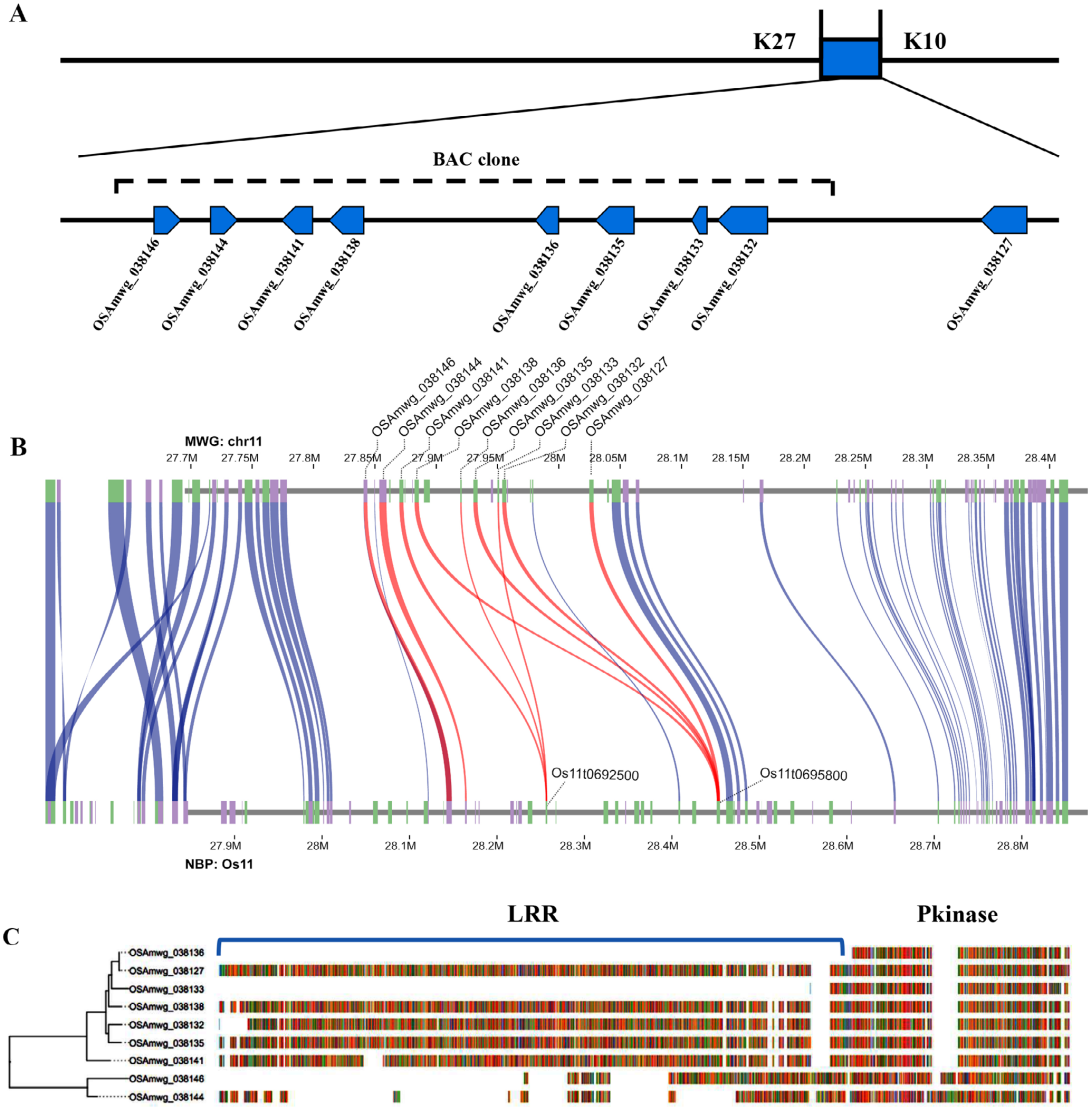
Overview of *Pi49* localization regions. (A) Schematic illustration of the candidate genes arrangement in *Pi49* localization regions. The direction of the arrow indicates the orientation of genes. The dashed line represents the BAC clone region. (B) Collinearity analyses of *Pi49* localization regions between Mowanggu (MWG) and Nipponbare (NPB). Red lines indicate homologous gene pairs of nine candidate genes. (C) Phylogenetic tree based on candidate amino acid sequences. The blue lines represent domains.

We identified nine QTLs (3.03–55.47 phenotypic contribution) associated with blast resistance distributed on chromosomes 2, 3, 6, 8, 9, 11 and 12, by using a recombinant inbred line F_8_ and F_9_ population derived from a cross between MWG and CO39 (Sun et al. 2013). Among them, *Pi49* was located between CAPS marker K27 and SSR marker K10 on chromosome 11. In the NPB genome, this interval spans a physical distance of 0.31 Mb and contains 34 annotated genes. The syntenic region in the MWG genome assembly is 0.19 Mb in length and contains 21 annotated genes. To evaluate the genetic variation in the *Pi49* localization region diversity across a range of rice varieties, we downloaded the 52 published rice genome sequences from NCBI and aligned their whole genome sequences with the MWG genome to calculate the coverage of each variety in *Pi49* localization regions. The majority of rice varieties did not contain it, except for 9311, Tetep, Co39, Shuhui498, R99, IRGC 26624‐2, IR64, A123 and Zhonghua11 (seven *indica* varieties and two *japonica* varieties) containing partial sequences of *Pi49* localization regions (coverage greater than 70%). Moreover, we searched the published *O. rufipogon* genome sequences but found no homologous sequence. Nine candidate genes in *Pi49* localization regions have the typical plant receptor kinase structure: OSAmwg_038132, OSAmwg_038133, OSAmwg_038135, OSAmwg_038136, OSAmwg_038138, OSAmwg_038141, OSAmwg_038144, OSAmwg_038146 and OSAmwg_038127. Of these, seven candidate genes (OSAmwg_038127, OSAmwg_038132, OSAmwg_038133, OSAmwg_038135, OSAmwg_038136, OSAmwg_038138, OSAmwg_038141) are on the same strand and two candidate genes (OSAmwg_038144, OSAmwg_038146) are on the same chromosome but not on the same strand (Figure 3A). Collinearity analyses of *Pi49* localization regions between MWG and NPB revealed that the two ends of the *Pi49* localization region were relatively conserved, and the gene collinearity was one‐to‐one correspondence, whereas the candidate kinase gene region had a high degree of variation. Except for OSAmwg_038144 and OSAmwg_038146, the remaining seven candidate genes may undergo gene duplication and have distinct biological functions (Figure 3B). The phylogenetic tree shows that these RLK genes are divided into two clusters: one with OSAmwg_038144 and OSAmwg_038146, and the other with the seven remaining candidate genes, which corresponds to the chromosomal orientation and indicates that the two branches arise from two different origins (Figure 3C). The seven RLK genes on the same branch are homologous genes that are presumed to have been duplicated by the same ancestral gene.

**FIGURE 4:**
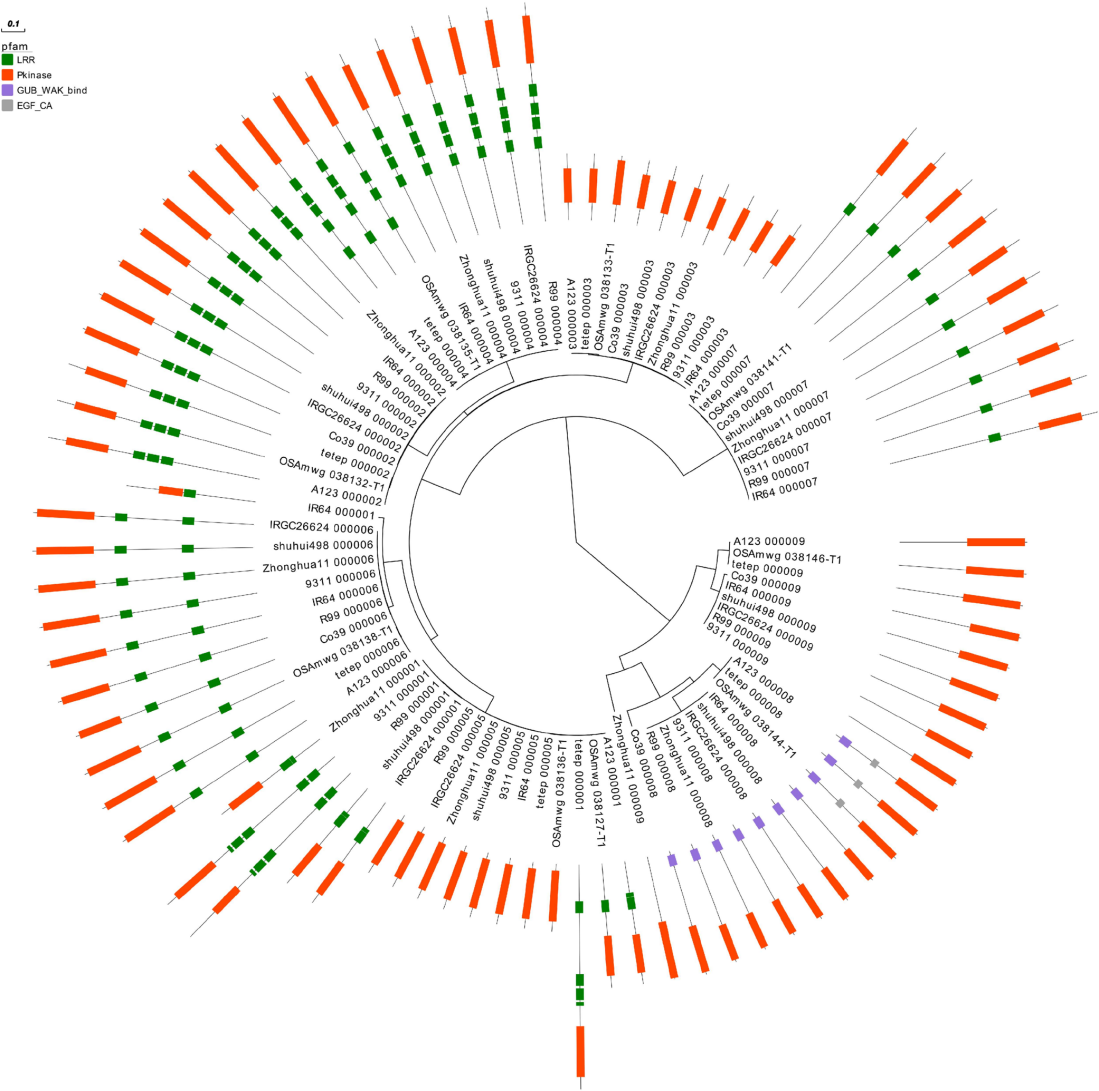
Comparative genome analysis of *Pi49* localization regions. The tree scale is 0.1 substitutions per site along the length specified. Different shapes and colours represent different conserved domains. The red rectangles represent the kinase domain. The green ovals represent the LRR domain. The blue rhombuses represent the WAK domain.

Through homology‐based searches, we predicted homologous genes from the genomes of other rice varieties that contain this region. According to the results, nine RLK genes were predicted from Tetep, Shuhui498, R99, IRGC 26624‐2, IR64 and Zhonghua11, eight RLK genes from 9311 and A123, six RLK genes from Co39, and five RLK genes were predicted from NPB. After multiple sequence alignment of the amino acid sequences of these RLK genes (a total of 81 RLK genes), the phylogenetic tree was constructed using IQTREE (Figure 4). The evolutionary tree divided these RLK genes into two clades, similar to the evolutionary tree constructed using only the RLK genes from the MWG. The orientation of the RLK gene arrangement within this region is conserved in other rice varieties, implying that they may have evolved from the same source. RLKs typically have two conserved domains: kinase and leucine‐rich repeat (LRR). Clade 1 members lack the LRR domain, while Clade 2 members, with the exception of a few, have both kinase and LRR domains.

### 2.5 OSAmwg_038136 Positively Contributes to Disease Resistance

**FIGURE 5:**
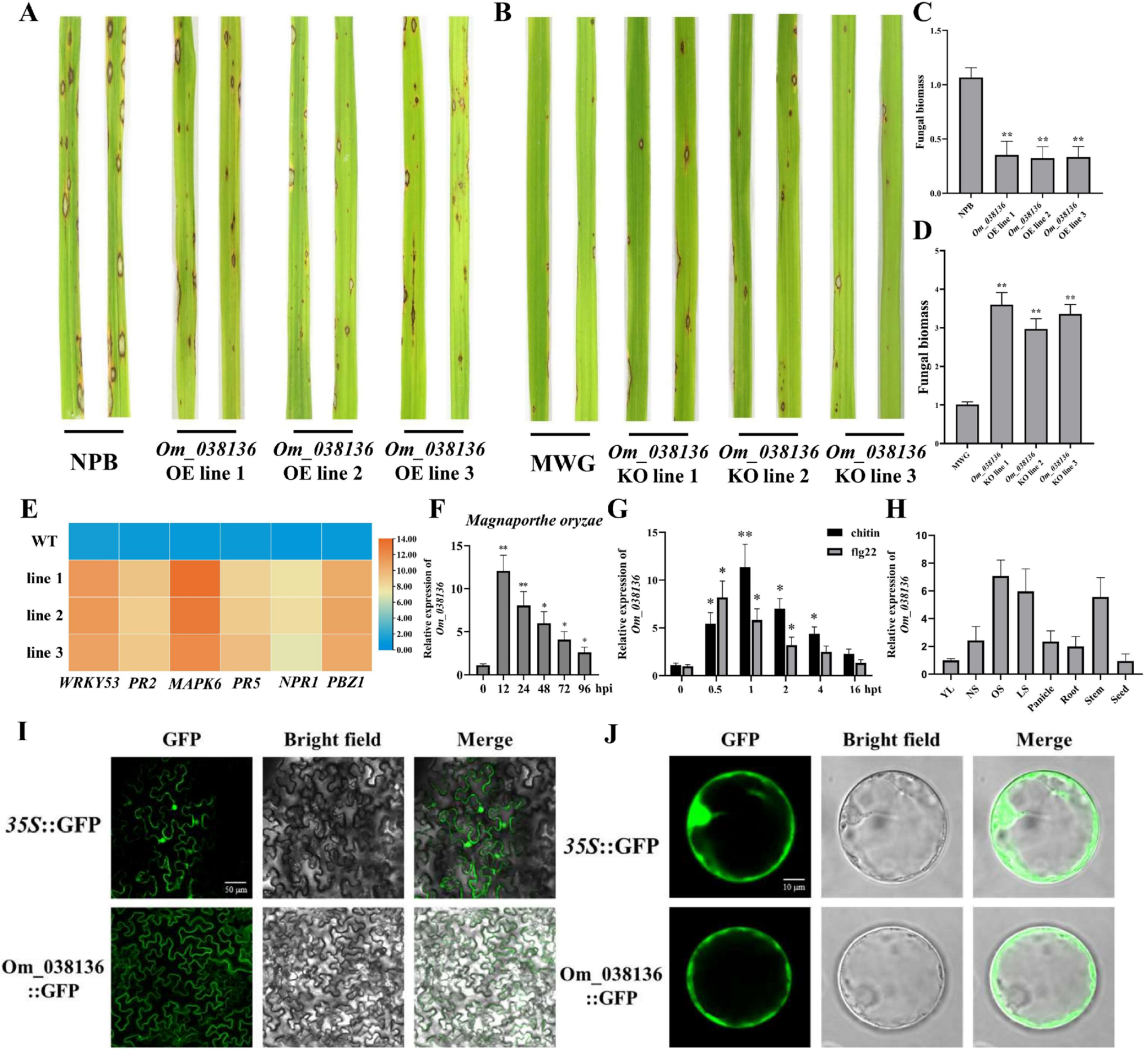
Om_038136 positively contributes to disease resistance. (A) Phenotypes of *Om_038136*‐overexpressing transgenic rice plants after inoculation with *Magnaporthe oryzae* in Nipponbare (NPB) background. (B) Phenotypes of *Om_038136* CRlSPR‐Cas9 knockout (KO) transgenic plants after inoculation with *M. oryzae* in Mowanggu (MWG) background. (C) Fungal biomass of *Om_038136*‐OE transgenic plants in NPB background. (D) Fungal biomass of *Om_038136*‐KO transgenic plants in MWG background. (E) Heat map of relative expression of *PR* genes in *Om_038136*‐OE transgenic plants after inoculation with *M. oryzae*. Orange colour represents low expression and blue colour represents high expression. (F) Relative expression of *Om_038136* after inoculation of MWG with *M. oryzae*. (G) Relative expression of Om_038136 after pathogen‐associated molecular pattern (PAMP) treatment in MWG. (H) Analysis of *Om_038136* expression patterns in different rice tissues. YL, young leaf (5 days before heading); NS, non‐senescent (14 days after heading); OS, onset of senescence (30 days after heading); and LS, late senescence (60 days after heading). (I) Subcellular localization of Om_038136 in *Nicotiana benthamiana* leaves. Bar = 50 μm. (J) Subcellular localization of Om_038136 in rice protoplasts. Bar = 10 μm. Relative gene expression levels were normalised using two internal reference genes, *OsUBQ* and *OsActin*. The data are the mean of three replicates ± SE. Asterisks denote statistically significant differences based on nested ANOVA (*p < 0.05, **p < 0.01).

**FIGURE 6:**
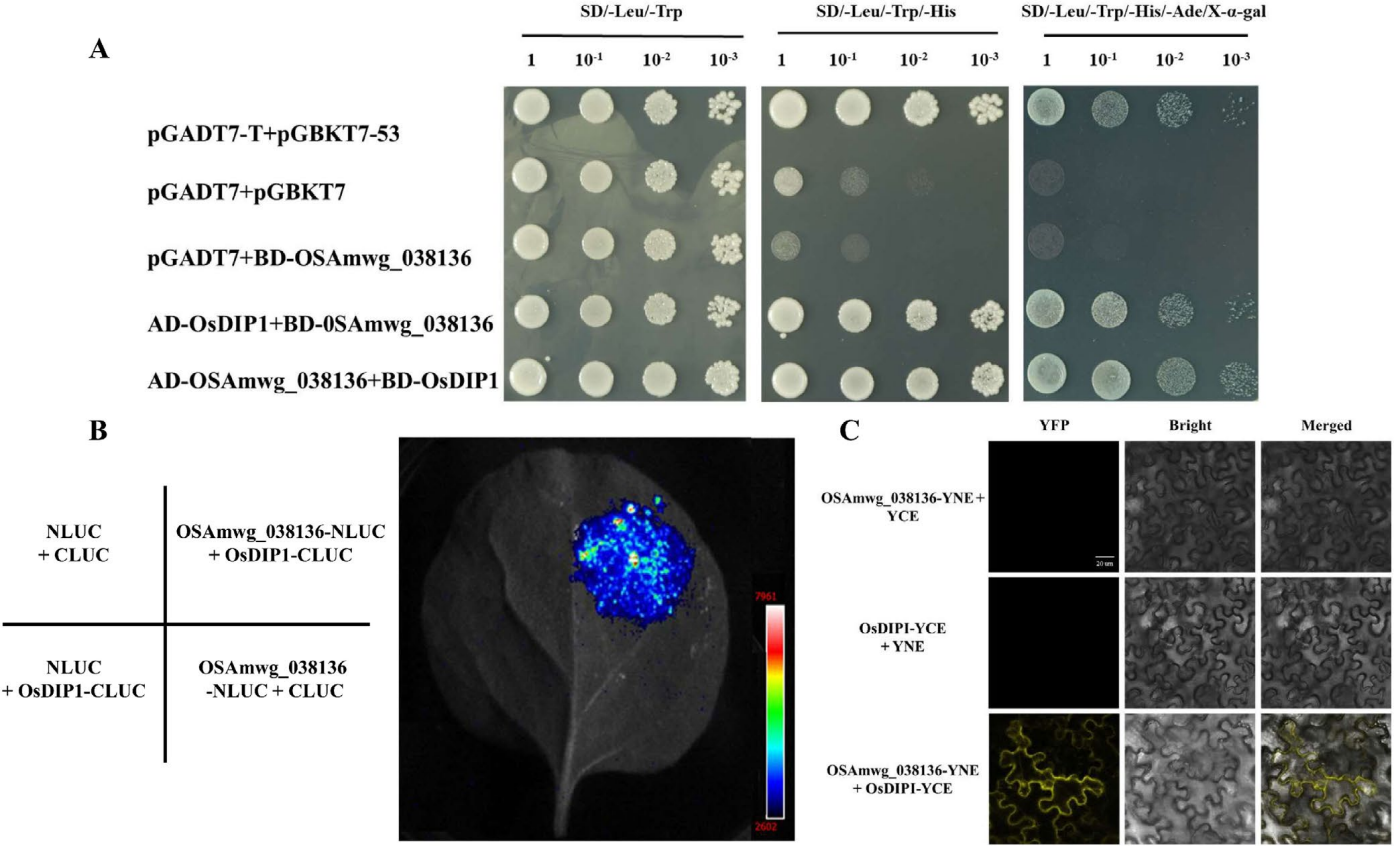
Verification of interaction between OSAmwg_038136 and OsDIP1. (A) Yeast two‐hybrid assay. Different selection media and various yeast concentrations are labelled above each image. (B) Luciferase complementation assay in *Nicotiana benthamiana* leaf. Each of the four quadrants was infiltrated with different constructs. The colour bar represents the fluorescence intensity. (C) Bimolecular fluorescence complementation in *N. benthamiana* leaf. From left to right in each image: fluorescence channel, bright field and overlay of fluorescence and bright‐field channels. Scale bars: 20 μm.

To investigate the roles of these candidates, full‐length cDNA for each candidate gene was independently cloned into an overexpression vector and obtained transgene‐positive plants in the NPB background. There were no obvious phenotypic differences between the *OSAmwg_038132*, *OSAmwg_038133*, *OSAmwg_038135*, *OSAmwg_038138*, *OSAmwg_038141*, *OSAmwg_038144*, *OSAmwg_038146*‐overexpressing plants and the NPB‐inoculated plants after spray inoculation with *M. oryzae* (Figure S5). In contrast, *OSAmwg_038136* (hereafter *Om_038136*)‐overexpressing plants reproducibly exhibited a significant improvement in disease resistance with fewer and smaller lesions than NPB background plants (Figure 5A). To corroborate findings from the overexpression lines, we also generated *Om_038136* knockout (*KO*) lines using CRISPR/Cas9 in the MWG background. In contrast to *Om_038136*‐OE plants, *Om_038136*‐KO plants showed more lesions after spray inoculation with *M. oryzae* relative to MWG (Figure 5B). To more precisely quantify the resistance phenotype, fungal biomass and lesions density were determined using quantitative PCR (qPCR) analysis and ImageJ software. We found that the fungal biomass of *M. oryzae* was much lower post‐inoculation in *Om_038136*‐OE plants and higher in *Om_038136*‐KO plants compared to the background plants (Figure 5C,D). In addition, the field phenotypes and phenotypic stability of the above results were verified in the Rice Blast Identification Center (Figure S6). Consistent with this, the relative expression levels of pathogenesis‐related (PR) genes, including *OsWRKY53*, *OsNPR1*, *OsPR2*, *OsRR5*, *OsMAPK6* and *OsPBZ1*, were higher in the *Om_038136*‐OE plants compared with the wild‐type background plants (Figure 5E). The differential expression pattern of *Om_038136* in MWG was profiled to gain insight into its biological function. As shown in Figure 6F, *Om_038136* expression peaked at 12 h and then gradually declined fo *M. oryzae* inoculation (Figure 5F). Because chitin and flg22 are strong elicitors of pathogen‐associated molecular pattern (PAMP)‐triggered immunity (PTI), the expression of *Om_038136* was analysed in flg22‐ and chitin‐treated plants. *Om_038136* exhibited a marked early response to flg22‐ and chitin‐induced PTI, as the relative expression of *Om_038136* increased quickly upon a perception of flg22 (reached the highest value at 0.5 h post‐treatment [hpt]) and chitin (reached the highest value at 1 hpt) and then gradually decreased (Figure 5G). *Om_038136* was highly expressed in the leaf and stem but not in the root and seed (Figure 5H). To investigate the precise subcellular localization of *Om_038136*, an Om_038136‐GFP fusion protein was transiently expressed in *Nicotiana benthamiana* leaves and an obvious fluorescent signal appeared specifically at the cell membrane (Figure 5I). In agreement with this finding, fluorescence from Om_038136‐GFP fusion protein was mainly localised in cell membrane in rice protoplasts (Figure 5J).

### 2.6 OSAmwg_038136 Interacts With OsDIP1

To gain a better understanding of the molecular mechanisms underlying Om‐038136 mediated blast resistance, we used a yeast two‐hybrid (Y2H) assay system with Om as bait to screen potential interacting proteins from a rice– *M. oryzae* cDNA library. The top hit of positive clones was a gene encoding a protein of 336 amino acids with an R3H domain, which corresponds to the previously reported protein OsDIP1 (Huang et al. 2022). The full‐length coding sequence was introduced into the bait and prey vectors to examine the relationship between Om and OsDIP1 (Figure 6A). The findings were validated *in planta* using multiple techniques. For instance, we verified Om_038136‐OsDIP1 interaction with an luciferase complementation imaging (LCI) assay in *N. benthamiana* leaves, and an intense luciferase signal was detected following co‐expression of Om_038136‐nLuc and OsDIP1‐cLuc (Figure 6B). Bimolecular fluorescence complementation (BiFC) assays confirmed the Om_038136–OsDIP1 interaction, which mirrored the LCI results (Figure 6C). These results collectively suggested that Om_038136 specifically interacts with OsDIP1 *in planta*.

**FIGURE 7:**
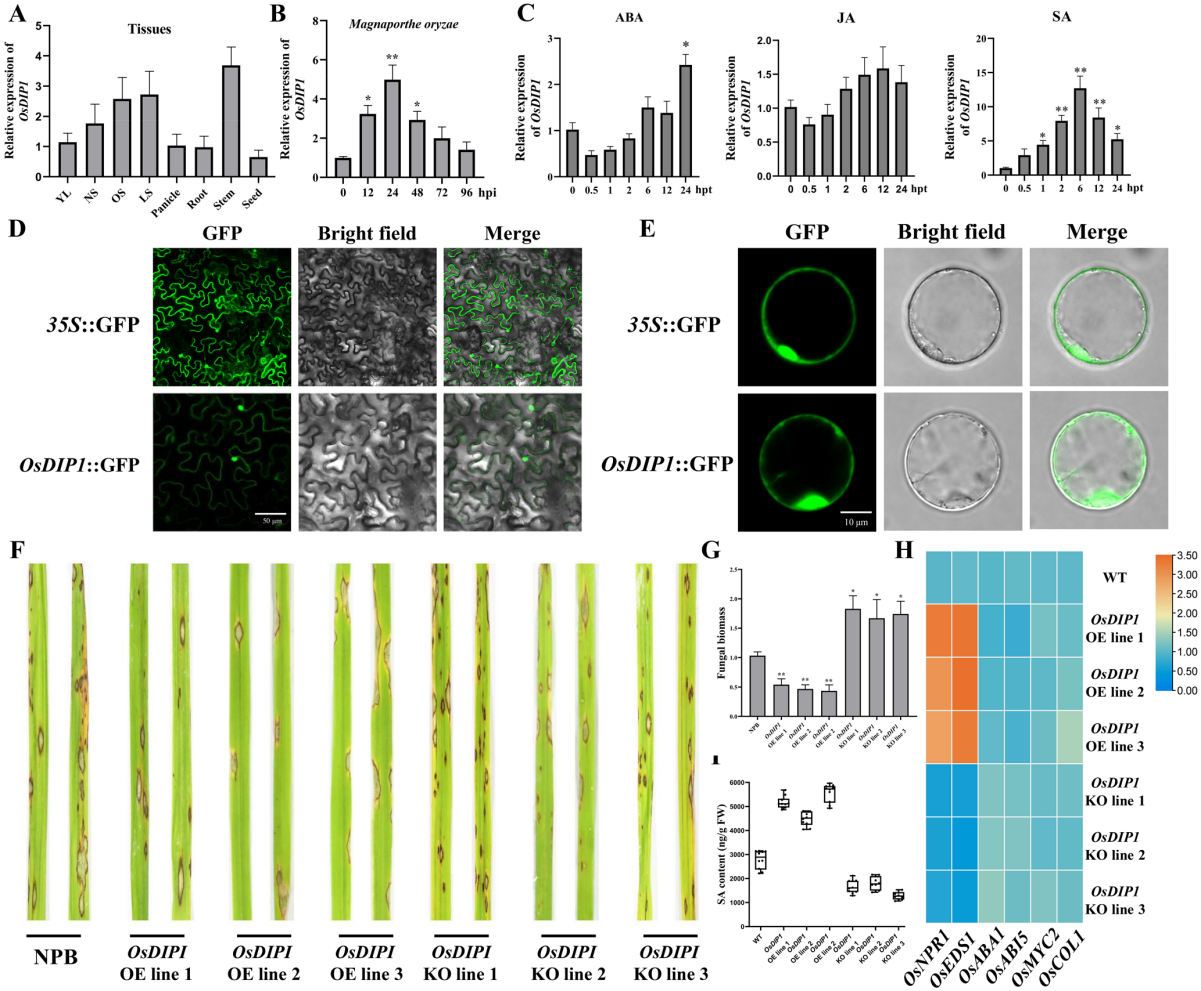
The role of OsDIP1 in resistance to rice blast. (A) Analysis of *OsDIP1* expression patterns in different tissues. YL, young leaf (5 days before heading); NS, non‐senescent (14 days after heading); OS, onset of senescence (30 days after heading); LS, late senescence (60 days afyter heading). (B) Relative expression of *OsDIP1* after inoculation with *Magnaporthe oryzae*. hpi, hours post‐inoculation. (C) Relative expression of *OsDIP1* after treatment with various hormones. ABA, abscisic acid; JA, jasmonic acid; SA, salicylic acid. (D) Subcellular localization of OsDIP1 in *Nicotiana benthamiana* leaves. Bar = 50 μm. (E) Subcellular localization of OsDIP1 in rice protoplasts. Bar = 10 μm. (F) Phenotype of *OsDIP1* CRlSPR‐Cas9 knockout (KO) and overexpression (OE) lines in Nipponbare (NPB) background after inoculation with *Magnaporthe oryzae*. (G) Fungal biomass of *OsDIP1* transgenic plants after inoculation with *M. oryzae*. (H) Heat map of hormone signalling pathways genes relative expression of *OsDIP1* transgenic plants after inoculation with *M. oryzae*. The expression of each gene in the wild type (WT) was used as a normalisation reference control. Orange colour represents low expression and blue colour represents high expression. (I) The content of SA of *OsDIP1*‐transgenic plants after inoculation with *M. oryzae*. Boxplots show minimum, maximum, and median, and each dot represents an independent biological replicate. Relative gene expression levels were normalised using two internal reference genes, *OsUBQ* and *OsActin*. The data are the mean of three replicates ± SE. Asterisks denote statistically significant differences based on nested ANOVA (*p < 0.05, **p < 0.01).

To determine whether OsDIP1 is involved in rice immunity, we used reverse transcription‐quantitative PCR (RT‐qPCR) to quantify *OsDIP1* mRNA levels in various tissues and under rice blast infection and different phytohormone treatments. The *OsDIP1* gene was widely expressed in the various tissues, but it was most abundant in the leaf (Figure 7A). *OsDIP1* responded to *M. oryzae* from an early stage (1 dpi), and then gradually decreased (Figure 7B). Previous studies have shown that OsDIP1 enhances tolerance to abiotic stresses via salicylic acid (SA) and abscisic acid (ABA) signalling pathways (Huang et al. 2022). To further explore OsDIP1's role in rice blast resistance and its interaction with these hormonal pathways, we conducted hormone treatment assays. After SA treatment, *OsDIP1* expression increased rapidly and peaked at 6 h post‐treatment (hpt). In contrast, there was no obvious effect in the relative expression of *OsDIP1* after jasmonic acid (JA) or ABA treatment (Figure 7C). Subcellular localization results revealed that OsDIP1 was localised in the cell nucleus and cell membrane in *N. benthamiana* (Figure 7D). In agreement with this finding, fluorescence from OsDIP1‐GFP fusion protein was mainly localised in the cell nucleus and cell membrane in rice protoplasts (Figure 7E). *OsDIP1* CRlSPR‐Cas9 knockout (KO) and overexpression (OE) plants were generated in the background of NPB to investigate the role of OsDIP1 in blast resistance. The results suggested that OsDIP1 positively regulates rice blast resistance (Figure 7F). This hypothesis was also supported by statistical data on fungal biomass (Figure 7G). Furthermore, the relative expression levels of SA pathway‐related genes (*NPR1* and *EDS1*) increased in *OsDIP1*‐OE plants after inoculation. In contrast, these genes showed a slight decrease in *OsDIP1*‐KO mutants. On the other hand, the expression levels of ABA biosynthesis‐related genes (*ABA1* and *ABI5*) and JA signalling‐related genes (*MYC2* and *COL1*) had no significant changes (Figure 7H). To go a step further, we measured the content of SA in transgenic and wild‐type plants (Figure 7I). The result showed that the content of SA increased significantly in *OsDIP1‐*OE and decreased in *OsDIP1*‐KO mutants.

## 3 Discussion

Sequence diversity is generally depicted by comparison with the reference genome; however, given the high genetic and phenotypic diversity between various modern cultivars (particularly rice), this method may be inadequate to distinguish highly polymorphic regions and cannot reveal the presence or absence of genes residing in exclusive (breed‐specific) regions of the genome (Li et al. 2024). In this view, whole‐genome sequencing and re‐annotation of various rice varieties with desirable traits is a resource‐intensive and indispensable task. In the current study, we sequenced the genome of durably resistant rice cultivar MWG using a combination of Illumina and Oxford Nanopore techniques. The final assembly genome size was 380.5 Mb, which is slightly less than the *indica* variety 9311 at 395.5 Mb but slightly larger than the NPB at 373.8 Mb (Zhang et al. 2022). The MWG genome assembly had few fragmented sequences, a small number of contigs with high N50, and a high level of complete BUSCO scores (98.2%), indicating a reasonably complete and high‐quality genome.

Collinearity analysis is an essential analytical strategy in comparative genomics because it provides valuable information about inter‐ and intraspecies conservation and variability. The MWG genome is densely packed with segmental duplications and has high collinearity with the NPB genome. In general, the length of the collinear sequence is inversely proportional to the species divergence time (Choi et al. 2020). Recently diverged species tend to accumulate less variation and retain higher structural and functional similarity, which is consistent with the species evolution analysis in our study. Different rice cultivars have undergone unique gene family expansions or contractions over evolutionary timescales, which are changes that underpin phenotypic evolution (Wu et al. 2020). Several expansions of lineage‐specific gene families in MWG are enriched for processes involved in the defence response, the response to biotic stimuli, and plant–pathogen interactions, implying that new functions to combat pathogenic challenges may be facilitated. All these analyses are in accordance with the high biotic stress resistance of MWG.

Based on our previous research, we investigated the role of regions of MWG‐specific high blast resistance contribution (Sun et al. 2013; Peng et al. 2023). We generated eight candidate gene transgenic plants in the NPB background and inoculation results revealed that only *Om_038136* positively regulated blast resistance. Our functional validation of *Om_038136* demonstrated its significant role in enhancing blast resistance, as evidenced by the upregulation of pathogenesis‐related genes and reduced fungal biomass in overexpression lines. However, the relationship between Om_038136 and the major resistance locus *Pi49* remains to be fully elucidated. According to the gene‐for‐gene hypothesis, the interaction between a plant resistance (*R*) gene and a pathogen avirulence (*Avr*) gene is essential for triggering a robust immune response (Kou et al. 2024). While *Om_038136* is located within the *Pi49* region and exhibits resistance‐related functions, we have not yet identified the corresponding avirulence gene in *M. oryzae* that interacts with *Om_038136*. This lack of direct evidence prevents us from conclusively assigning *Om_038136* as the *Pi49* gene. Future studies should focus on identifying the avirulence gene in *M. oryzae* that specifically interacts with *Om_038136*, as this would provide critical evidence to support its role as the *Pi49* gene. Additionally, further functional analyses, such as allelic variation studies and complementation tests, are needed to confirm whether *Om_038136* is indeed the *Pi49* gene or if other genes within the *Pi49* locus contribute to the observed resistance phenotype.

Comparative genome analysis revealed that the *Pi49* localization regions were only present in a small number in the genomes of *indica* and *japonica* varieties, but not in *O. rufipogon* . In addition, a conserved sequence alignment of *Pi49* localization regions between MWG and six rice cultivars revealed that all six orthologous genes of *Om_038136* are from blast‐resistant varieties and have one amino acid substitution except for Tetep (Figure S7). Previous research has shown that ZFP36, OsDIP1 and OsDBF1 can form a trimer, which potentially enhances the stress resistance function of OsDBF1 (Huang et al. 2022). Six of the seven genes in the same orientation in *Pi49* localization regions formed three groups of pairwise genes. OsDIP1 was found to interact with Om_038136 and OSAmwg_038135 (results not shown). It is unknown whether OsDIP1, Om_038136 and OSAmwg_038135 form a trimer, resulting in increased immune responses and resistance to *M. oryzae* .

Om_038136 was found on the cell membrane and had the typical plant receptor kinase structure. The cell membrane is a crucial site for rice– *M. oryzae* biomolecular recognition and the expression or functional changes of membrane‐localised proteins are tightly linked to the plant immune response. For example, both the chitin elicitor receptor CEBiP and receptor‐like protein kinase Pi‐d2 located on the cell membrane form a complex, resulting in enhanced recognition of rice to chitin and improved resistance to rice blast (Kouzai et al. 2013). Similarly, *Om_038136* responds rapidly to *M. oryzae* inoculation and elicitor treatment, implying that Om_038136 might be involved in pathogen recognition or immune signalling. Of note, because *Om_038136* was expressed primarily in leaf and stem, we believe it may be involved in leaf blast and panicle blast resistance, but this finding warrants further validation. These data demonstrated the potential for breeding application and generalizability of Om_038136.

Moreover, using a Y2H screen, we discovered that OsDIP1 interacts with Om_038136. The interaction was verified using LCI and BiFC methods, and their subcellular localization suggested that the two proteins may have temporal and spatial opportunities to interact *in planta*. OsDIP1, an R3H protein family member with R3H and SUZ domains, is an RNA/DNA binding protein with multiple functions. ZmDIP1, an OsDIP1 homologous protein, was found to be differentially expressed in juvenile and adult foliar leaves of maize inbred lines. Transient expression in maize leaves revealed a close relationship between ZmDIP1‐mediated ROS and ABA signalling pathways and maize resistance to *Curvularia lunata* infection (Zhu et al. 2012). Likewise, the current study discovered that *OsDIP1* and *Om_038136* were highly expressed in leaves and may play important roles in leaves. Previous research has demonstrated that OsDIP1 regulates abiotic stress (low temperature, drought and high salt) tolerance in rice via the SA‐dependent signalling pathway (Huang et al. 2022). Consistent with this, we discovered that SA induced *OsDIP1* expression and the expression levels of SA pathway‐related genes and SA content were significantly higher in *OsDIP1*‐OE plants than in the wild type. This finding suggests that OsDIP1 plays a role in the SA‐mediated defence response of rice.

In conclusion, the availability of MWG‐quality genome sequences greatly expands our understanding of rice genome diversity, particularly that of traditional landraces. Furthermore, this research expands and enriches the genetic information reservoir for addressing a series of unresolved questions in plant bioinformatics and plant pathology including disease resistance, yield and quality. These sequences will also be significantly useful for rice biology and genetic improvement, which will help in meeting the increasingly global food requirements of humans.

## 4 Experimental Procedures

### 4.1 Plant Material and Growth Condition

The susceptible *japonica* cultivar NPB and the resistant *japonica* cultivar MWG were used in this study. Transgenic rice plants were generated via *Agrobacterium*‐mediated transformation. The target gene was cloned into the overexpression vector pXGUS‐P (with 35S promoter) and CRISPR/Cas9 vector pHEE402 (with UBI promoter), validated by XbaI/KpnI digestion and sequencing. Recombinant plasmids were electroporated into *Agrobacterium tumefaciens* EHA105, which was used to infect embryogenic calli derived from mature embryos. After co‐cultivation with 200 μM acetosyringone (3 days in darkness at 28°C), calli were selected on N6D2 medium containing 50 mg/L hygromycin B for 4 weeks. Regenerated plants were screened by droplet digital PCR (Bio‐Rad QX200) for transgene copy number. T_2_ homozygous lines with single‐copy insertions were propagated for phenotypic stability evaluation over three generations. Rice seeds were surface‐disinfected with 75% ethanol for 1–2 min and then presoaked in sterile distilled water for germination at 28°C ± 1°C for 4 days and the water was exchanged daily. Germinating seeds were picked and planted in seedling plug trays filled with a mixture of nutrient soil and vermiculite under normal greenhouse conditions of a photoperiod of 16‐h light and 8‐h dark and a humidity > 70% at 28°C ± 1°C. Rice seedlings were transferred to a constant temperature and humidity incubator (28°C ± 1°C, 90% RH) and used for inoculation with a highly pathogenic *M. oryzae* strain (110‐2).

### 4.2 Fungal Inoculation

Cultivation and inoculation of *M. oryzae* strain 110‐2 were performed according to the method described previously with minor modifications (Peng et al. 2021). The strain 110‐2 was cultured at 28°C ± 1°C on tomato‐oatmeal medium (tomato extract 350 mL, oatmeal 30 g, CaCO_3_ 0.5 g, agar 15 g and distilled water 1 L) for 2 weeks with 12‐h light and 12‐h dark cycles. Fresh conidia on the surface of the tomato‐oatmeal medium were rinsed off by distilled water with 0.02% Tween 20 and filtered through two layers of surgical gauze. For spray‐inoculation, the spore suspension was adjusted to a final concentration at 5 × 10^5^ conidia/mL with distilled water and sprayed evenly on the leaves of three‐leaf‐stage rice seedlings (approximately 20 days old) while the control group was mock‐inoculated with sterile 0.02% Tween 20 only. Inoculated plants were raised in the dark with high humidity (> 90%) for 1 day and then transferred into normal culture conditions in humidified incubators for 5 days for disease development.

### 4.3 Genome Sequencing and Assembly

Genomic DNA was extracted following the CTAB method, concentration and purity of the DNA was determined using UNano‐3000 micronuclei acid analyser (Zhouming Instruments). The purified genomic DNA was used to construct nanopore sequencing library by using SQK‐LSK109 (Oxford Nanopore Technologies) according to the manufacturer's instructions, and Oxford Nanopore Technology sequencer Nanopore PromethION was used for DNA single‐molecule sequencing to obtain the sequencing data, which were carried out in GrandOmics Biosciences Co. Ltd. The Nanopore raw reads were preprocessed using Porechop (v. 0.2.4) for adapter removal. Initial quality assessment was performed with NanoPlot (v. 1.28.0). Quality filtering was conducted using Filtlong (v. 0.2.1) with the following parameters: a minimum mean quality score of 7 and a minimum read length of 1 kb. After filtering, the dataset had an N50 of 28 kb. The resulting high‐quality reads were de novoassembled with NextDenovo v. 2.5.0. The assembly was subsequently polished using NextPolish v. 1.4.1 (Hu et al. 2020) with both long and short reads, followed by two additional rounds of polishing with Racon (Vaser et al. 2017) to generate the final assembly. The completeness of the genome assembly was evaluated with BUSCO v. 5.4.3 (Manni et al. 2021) using both genome and annotated protein modes. Representative proteins from 14 plant genomes, *Oryza barthii* , *Oryza glaberrima* , *Oryza nivara*, *Oryza rufipogon* , *Oryza sativa* subsp. *indica*, *O. sativa* subsp. *japonica* (MWG), *Oryza glumipatula* , *Oryza longistaminata* , *Oryza meridionalis*, *Oryza punctata* , *Oryza brachyantha* and *Zea mays* were clustered into orthogroups (OGs) based on sequence similarity using OrthoFinder v. 2.4.0 (Emms and Kelly 2019). CAFÉ v. 5.0 (Mendes et al. 2021) was used to analyse the expansion and contraction of gene families. The species tree was generated using IQ‐TREE v. 2.2.0 (Nguyen et al. 2015) based on the single‐copy gene families identified by OrthoFinder. With the species tree, r8s (Sanderson 2003) was employed to estimate the divergence time of each branch. The divergence times of *O. sativa* and *Z. mays* (62 MYA) from the TimeTree database (Kumar et al. 2017) were used as references.

### 4.4 Genome Annotation, Comparative Analysis and Resistance Gene Prediction

A strategy combining ab initio gene prediction, homology‐based gene prediction and RNA‐seq was used for gene annotation. The repetitive sequences were annotated by combining ab initio and homology‐based methods. First, ab initio repeat library was predicted with RepeatModeler v. 2.0.14 (Flynn et al. 2020). Second, this library was combined with Repbase v. 28.02 (http://www.girinst.org/repbase) for identifying all homologous repeats throughout the genome by RepeatMasker v. 4.1.4 (Chen 2004) (http://www.repeatmasker.org/) using WU‐BLASTX as the search engine. The RNA‐seq reads were assembled into contigs using Trinity v. 2.8.6 (Grabherr et al. 2011) with default parameters and further predicted gene structures using PASA pipeline v. 2.5.1 (Haas 2003). We trained Augustus (Stanke et al. 2006) and SNAP using the highly confident gene models from the results of PASA assembly, while GeneMark‐ES (Lomsadze et al. 2014) was self‐trained on the repeat‐masked genome sequences. Homologous protein sequences from *Arabidopsis thaliana* (TAIR10), *O. sativa* subsp. *japonica* (IRGSP‐1.0) and *O. sativa* subsp. *indica* (Ensembl 48) were downloaded from EnsemblPlants (https://plants.ensembl.org/), and all of these sequences were mapped to each assembly with tBLASTN with an *E*‐value cut‐off of 1e−5. Genewise (Birney et al. 2004) (parameter: ‐gff ‐quiet ‐silent ‐sum) was used to refine the alignment. All results were integrated into consensus gene models using EvidenceModeler (Haas et al. 2008). To identify resistance (*R*) genes, we used RGAugury (Li et al. 2016) to scan the whole‐genome annotation and classify candidate *R* genes into subgroups, such as NBS, RLK, RLP and others. Synteny and collinearity analysis was performed using MCScanX. Protein sequences from both genomes were aligned using BLASTP with an *E*‐value cut‐off of 1e−10. Homologous gene pairs were identified with the following MCScanX parameters: match_size = 5 for all‐vs‐all BLASTP, max_gaps = 10 genes and *p*value_cutoff = 1e−10. Segmental duplications were defined as collinear blocks containing ≥ 5 gene pairs. Syntenic relationships were visualised using Circos v. 0.69 (Wang, Tang, et al. 2024).

### 4.5 RNA Isolation and RT‐qPCR Analysis

Total RNA extraction method was used to extract RNA from the fresh rice leaves as described previously (Peng et al. 2023). The concentration and purity of RNA were checked by using plant NanoPhotometer spectrophotometer (IMPLEN) and the RNA quality was further assessed with a Bioanalyzer RNA pico kit (Agilent Technologies). Reverse transcription was performed using QuantiTect Reverse Transcription Kit (QIAGEN), and qPCR analysis was conducted using SYBR qPCR MagicMix (Noble Ryder Technology). Gene expression levels were normalised to *OsUBQ* and *OsActin* (internal control) and quantified using the 2^−ΔΔCt^ method (Maren et al. 2023). Target genes were analysed only if their *C*_t_ values fell within the validated dynamic range (15–35 cycles), ensuring amplification efficiency comparable to the reference gene. The reaction procedure was performed according to the kit instructions. All qPCR primers were designed by Oligo v. 7.56 and sequences are provided in Table S1. Statistical significance of differences between group means was evaluated using unpaired *t*‐tests or one‐way ANOVA with appropriate post hoc tests, as indicated in the figure legends. All analyses were performed with GraphPad Prism, v. 10.3.0 (Mitteer et al. 2018). All experiments were performed with three biological replicates.

### 4.6 PAMP Treatments and HPLC‐MS Analysis

The mature healthy rice leaves were sliced into small leaf disks (1 cm^2^) and imbibed in distilled water overnight at room temperature. Three pieces of each line were put into a 2‐mL sterile tube filled with 80 nM flg22 or 10 nM chitin. Samples were taken at 0 h (experiment initiation), 0.5,1, 2, 4 and 16 h and flash‐frozen in liquid nitrogen for storage. For hormone treatments, 1‐month‐old rice plants were sprayed with JA (100 μM, SA) (100 μM), ABA (100 μM), or sterile water (mock treatment). Following treatment, rice plants were transferred in a constant temperature and humidity incubator (28°C ± 1°C, 80% RH) and sampled at 0 h (experiment initiation), 0.5, 1, 2, 6, 12 and 24 hpt. To measure hormone contents, approximately 0.6 g of rice leaf tissue was placed in a steriled centrifuge tube, and 6 mL of acetonitrile was added. The tissue was thoroughly homogenised using a handheld homogeniser, followed by vortex mixing. The mixture was then subjected to ultrasonic disruption for 30 min. After high‐speed centrifugation at 5000 *g* for 10 min at 4°C, the supernatant was transferred to a new centrifuge tube. To remove pigments and impurities, 30 mg of graphitized carbon black (GCB) was added to the supernatant. The mixture was centrifuged again under high‐speed and low‐temperature conditions. The clarified supernatant was carefully aspirated using a syringe and filtered through a 0.22 μm organic filter membrane for subsequent analysis via high‐performance liquid chromatography‐mass spectrometry (HPLC‐MS). Chromatographic separation was achieved on a C18 reversed‐phase column (2.1 mm × 100 mm, 1.8 μm) maintained at 40°C. The mobile phase consisted of (A) 0.1% formic acid in water and (B) acetonitrile, using a gradient elution programme at a flow rate of 0.3 mL/min. Mass spectrometric detection was operated in multiple reaction monitoring (MRM) mode with electrospray ionisation (ESI) in negative polarity. Quantification was performed using external standard curves calibrated for each analyte (SA, JA, ABA). The hormone contents were normalised to the fresh weight of the tissue sample.

### 4.7 Subcellular Localization

Full‐length DNA fragments of target genes were constructed into a linearized pSuper1307 vector (digested with BamHI and SalI) between a cauliflower mosaic virus 35S promoter and green fluorescent protein (GFP) tag using the Seamless Cloning Kit (Beyotime). The recombinant pSuper1307 vectors (either containing the gene of interest or empty vector as a control) were introduced into *A. tumefaciens* LBA4404 by electroporation. Positive transformants were selected on YEP solid medium containing 50 μg/mL kanamycin, 50 μg/mL rifampicin, and 25 μg/mL streptomycin, and incubated at 28°C for 48 h. A single colony was inoculated into 5 mL of YEP liquid medium with the corresponding antibiotics and grown overnight at 28°C with shaking at 220 rpm. The bacterial culture was then centrifuged at 5000 *g* for 10 min at room temperature. The pellet was resuspended and diluted in infiltration buffer (10 mM MES, 10 mM MgCl₂, 150 μM acetosyringone, pH 5.6) to a final OD_600_ of 0.5. The bacterial suspension was kept at room temperature in the dark for 2–4 h without shaking. The abaxial side of 20‐day‐old *N. benthamiana* leaves was infiltrated using a 1 mL needleless syringe. For co‐infiltration experiments, equal volumes of different bacterial suspensions were mixed prior to infiltration. After infiltration, plants were kept under normal growth conditions (16 h light/8 h dark photoperiod, 25°C, 70% humidity) for 48–72 h before subsequent analysis.

The leaves were covered and examined using an Eclipse TE2000‐E confocal laser‐scanning microscope (Nikon) at 2 days post‐injection. For subcellular localization in rice protoplasts, etiolated rice seedlings grown in the dark for 10–15 days were used for protoplast isolation. The leaf sheaths were immersed in 0.6 M mannitol and rapidly cut into segments smaller than 1 mm using a disposable medical blade. The segments were then transferred into a prepared enzyme solution containing Cellulase R‐10 and Macerozyme R‐10. After vacuum infiltration for 5–10 min, the samples were incubated on a shaker at 28°C and 40 rpm for 4 h for enzymatic digestion. The digested solution was filtered through Miracloth, and the filtrate was collected in a 50 mL centrifuge tube. The protoplasts were collected by centrifugation at 100 *g* for 10 min at 4°C using prechilled MMG (400 mM mannitol, 15 mM MgCl₂, 40 mM MES, pH 5.6 adjusted with KOH) and W5 (154 mM NaCl, 125 mM CaCl₂, 5 mM KCl, 5 mM glucose, pH 5.8 adjusted with KOH) solutions. The protoplasts were gently resuspended and kept on ice for subsequent use. For transfection, 200 μL of protoplast suspension was mixed with 220 μL of 40% PEG 4000 solution in a 2 mL centrifuge tube. The mixture was immediately and gently inverted to ensure thorough mixing, and the timer was started to begin timing the 15 min incubation period. The expression of the fusion protein fluorescence was observed under a laser scanning confocal microscope to confirm the subcellular localization signal.

### 4.8 Validation of Protein Interactions

The coding sequences of proteins were cloned into pGADT7 (bait) and pGBKT7 (prey) vectors for Y2H experiments. The Alkali‐Cation Yeast Transformation Kit (Bio 101) was used to screen yeast libraries and for co‐transformation. The constructed plasmid, pGBKT7‐53/pGADT7‐T (positive control) and pGBKT7/pGADT7 (negative control) were individually transformed into yeast strain AH109 (WeiDi). The transformed AH109 yeast was homogeneously coated on the corresponding selection medium and incubated at 28°C for 3 days. LCI experiments were performed using a modified version of a previously reported method. The constructs were cloned into the pCAMBIA1300‐nLUC and pCAMBIA1300‐cLUC vectors and electroporated into *A. tumefaciens* LBA4404. The experimental or control groups (empty vector) was mixed with an equal volume of the P19 suppressor and then injected into the leaf epidermis of *N. benthamiana*. The injected leaves were smeared evenly with 4 mm potassium D‐luciferin for fluorescence detection 3 days later, and fluorescence imaging was performed using a Lumazone pylon 2048B bioluminescence imaging system.

## Author Contributions

Weiye Peng, Pingyong Sun, Nuan Yi, Zhuozhi Zheng, Jing Liu and Xionglun Liu performed the experiments and analysed the data. Bing Wang and Wei Li conceived and designed the experiments. Liangying Dai and Yunsheng Wang supervised the study, provided critical feedback, and contributed to the manuscript writing. All authors reviewed and approved the final version of the manuscript.

## Funding

This work was supported by the National Natural Science Foundation of China (31371246, 32272559), Agricultural Science and Technology Innovation Program of Hunan Province (2024CX099).

## Conflicts of Interest

The authors declare no conflicts of interest.

## Supporting information


**Figure S1:** Overview of the MWG genomes. Gene features across the MWG chromosomes. Tracks 0–5 represent the following: (0) genomic positions (in Mb) of the 12 MWG chromosomes; (1) GC content; (2) gene density; (3) resistance gene analogs; (4) genomic regions similar to japonica varieties; and (5) genomic regions similar to indica varieties. The window size is 200 kb.

**Figure S2:** Whole‐genome analysis of MWG (A) Segmental duplications analyses of the MWG genomes. (B) Collinearity analyses of the MWG and the NPB genomes.

**Figure S3:** Overview of the structural variations in the MWG genome. The lines in blue, red and green denote the NPB, MWG and CO39 genomes, respectively. The areas in grey, orange and yellow represent the syntenic region, inversion region and translocation region, respectively. Red squares denote the centromere and blue squares represent the *Pi49* localization region.

**Figure S4:** Phylogenetic tree of the RLK (A) and NB‐ARC (B) domain gene family identified in the MWG genome and NPB genome.

**Figure S5:** Phenotype of candidate genes in transgenic rice inoculated with *Magnaporthe oryzae*. The experiment was repeated with consistent results.

**Figure S6:** Field resistance evaluations under natural infection conditions. The field site was operated by an experimental field of the Rice Blast Identification Center in Taojiang, Hunan, China (112°06′34″ E, 28°38′55″ N).

**Figure S7:** Conserved sequence alignment of OSAmwg_038136 and orthologous genes between MWG and six rice cultivars. The grey‐shaded regions indicate identical amino acids among the different sequences, whereas the black‐shaded regions represent conserved replacements.

**Table S1:** qPCR primers in this study.

**Table S2:** Statistics of the assembly features for the MWG and other rice reference genomes.

## Data Availability

All data supporting the findings of this study are available within the paper and within its Supporting Information published online.
