# Supplementary figures and images for: The Genome of the Rice Variety Mowanggu Provides Insight Into Resistance to Magnaporthe oryzae
Source: Mol Plant Pathol. 2026 Mar 26;27(3):e70223. doi: 10.1111/mpp.70223 (PMC13097360; doi:10.1111/mpp.70223)

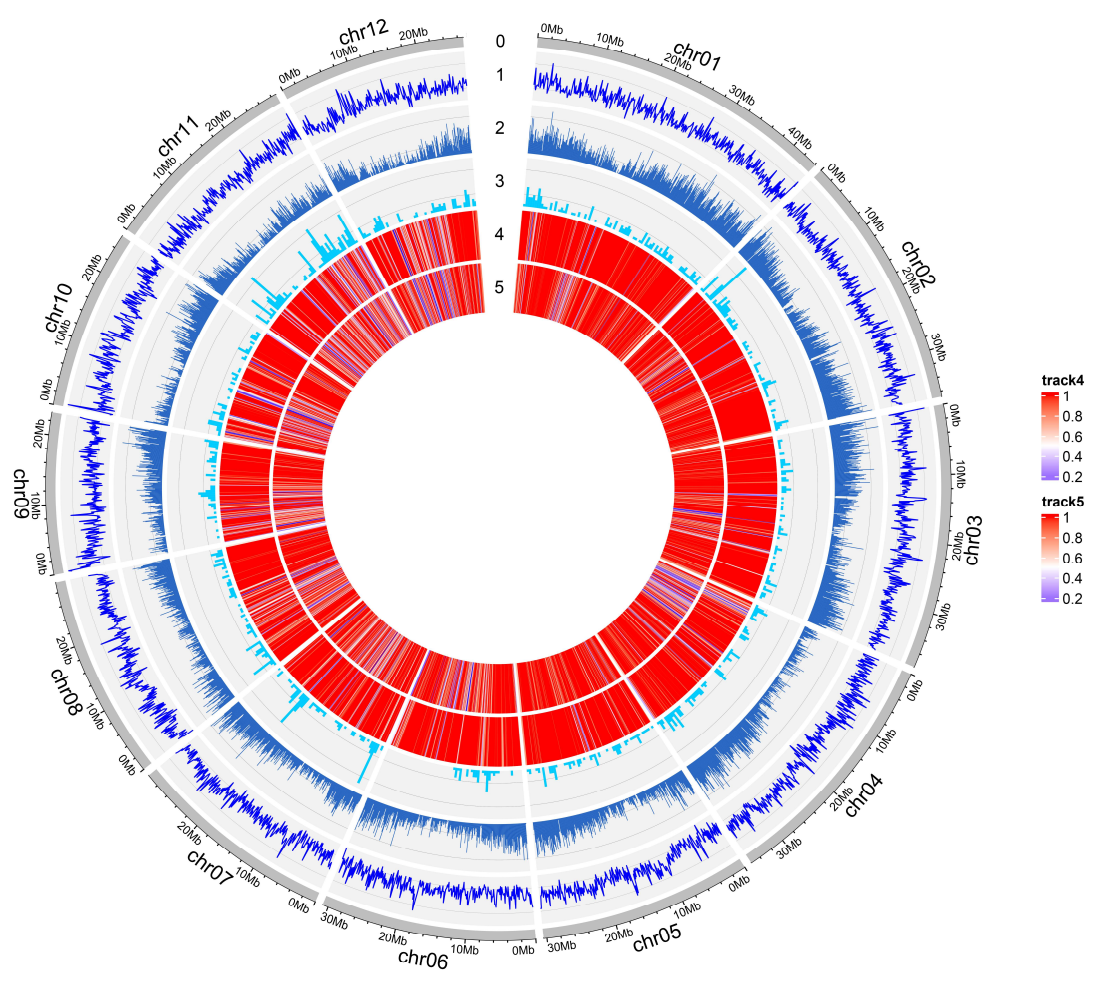

Supplement: Supplementary file 1 — Figure S1: Overview of the MWG genomes. Gene features across the MWG chromosomes. Tracks 0–5 represent the following: (0) genomic positions (in Mb) of the 12 MWG chromosomes; (1) GC content; (2) gene density; (3) resistance gene analogs; (4) genomic regions similar to japonica varieties; and (5) genomic regions similar to indica varieties. The window size is 200 kb. [file MPP-27-e70223-s005.pdf]

**A**

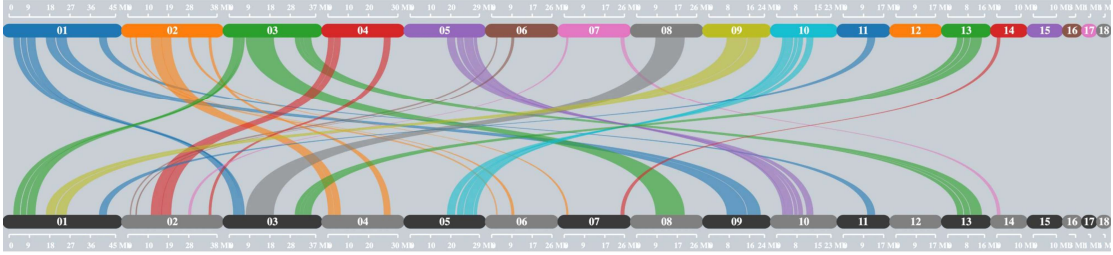

**B**

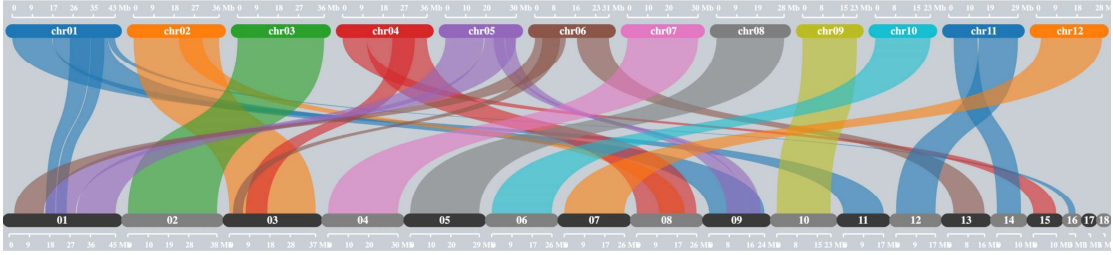

Supplement: Supplementary file 2 — Figure S2: Whole‐genome analysis of MWG (A) Segmental duplications analyses of the MWG genomes. (B) Collinearity analyses of the MWG and the NPB genomes. [file MPP-27-e70223-s007.pdf]

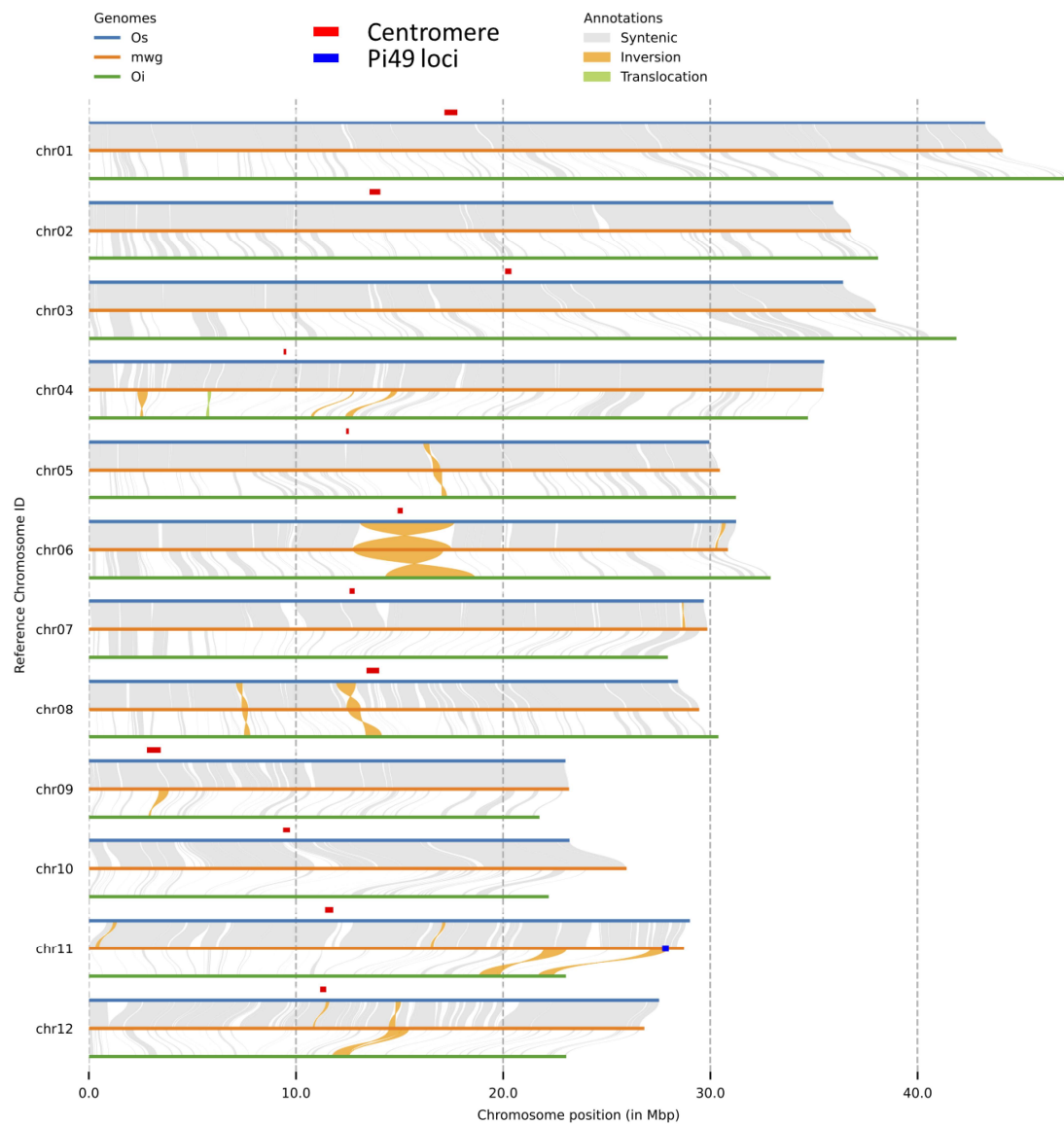

Supplement: Supplementary file 3 — Figure S3: Overview of the structural variations in the MWG genome. The lines in blue, red and green denote the NPB, MWG and CO39 genomes, respectively. The areas in grey, orange and yellow represent the syntenic region, inversion region and translocation region, respectively. Red squares denote the centromere and blue squares represent the Pi49 localization region. [file MPP-27-e70223-s008.pdf]

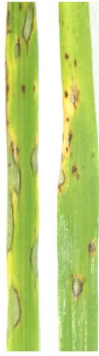

**WT**

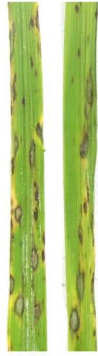

**OSAmwg\_  
038132-OE**

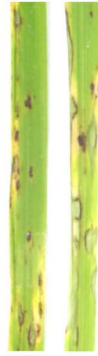

**OSAmwg\_  
038133-OE**

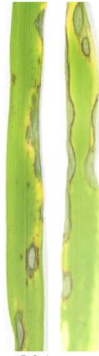

**OSAmwg\_  
038135-OE**

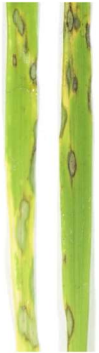

**OSAmwg\_  
038138-OE**

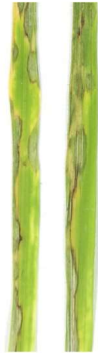

**OSAmwg\_  
038141-OE**

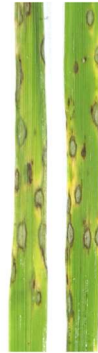

**OSAmwg\_  
038144-OE**

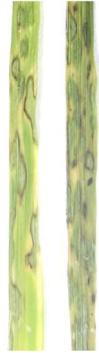

**OSAmwg\_  
038146-OE**

Supplement: Supplementary file 5 — Figure S5: Phenotype of candidate genes in transgenic rice inoculated with Magnaporthe oryzae. The experiment was repeated with consistent results. [file MPP-27-e70223-s002.pdf]

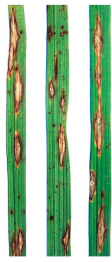

**NPB**

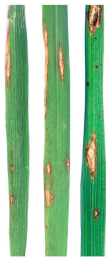

*Om\_038136*  
OE line 1

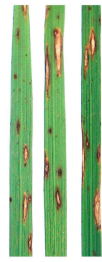

*Om\_038136*  
OE line 2

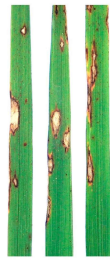

*Om\_038136*  
OE line 3

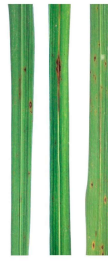

**MWG**

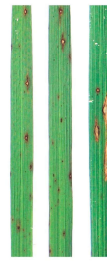

*Om\_038136*  
KO line 1

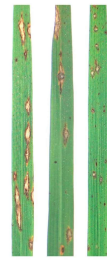

*Om\_038136*  
KO line 2

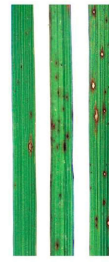

*Om\_038136*  
KO line 3

Supplement: Supplementary file 6 — Figure S6: Field resistance evaluations under natural infection conditions. The field site was operated by an experimental field of the Rice Blast Identification Center in Taojiang, Hunan, China (112°06′34″ E, 28°38′55″ N). [file MPP-27-e70223-s001.pdf]
