# Supplementary material for: The Genome of the Rice Variety Mowanggu Provides Insight Into Resistance to Magnaporthe oryzae
Source: Mol Plant Pathol. 2026 Mar 26;27(3):e70223. doi: 10.1111/mpp.70223 (PMC13097360; doi:10.1111/mpp.70223)
Supplement: Supplementary file 4 — Figure S4: Phylogenetic tree of the RLK (A) and NB‐ARC (B) domain gene family identified in the MWG genome and NPB genome. [file MPP-27-e70223-s004.pdf]

A

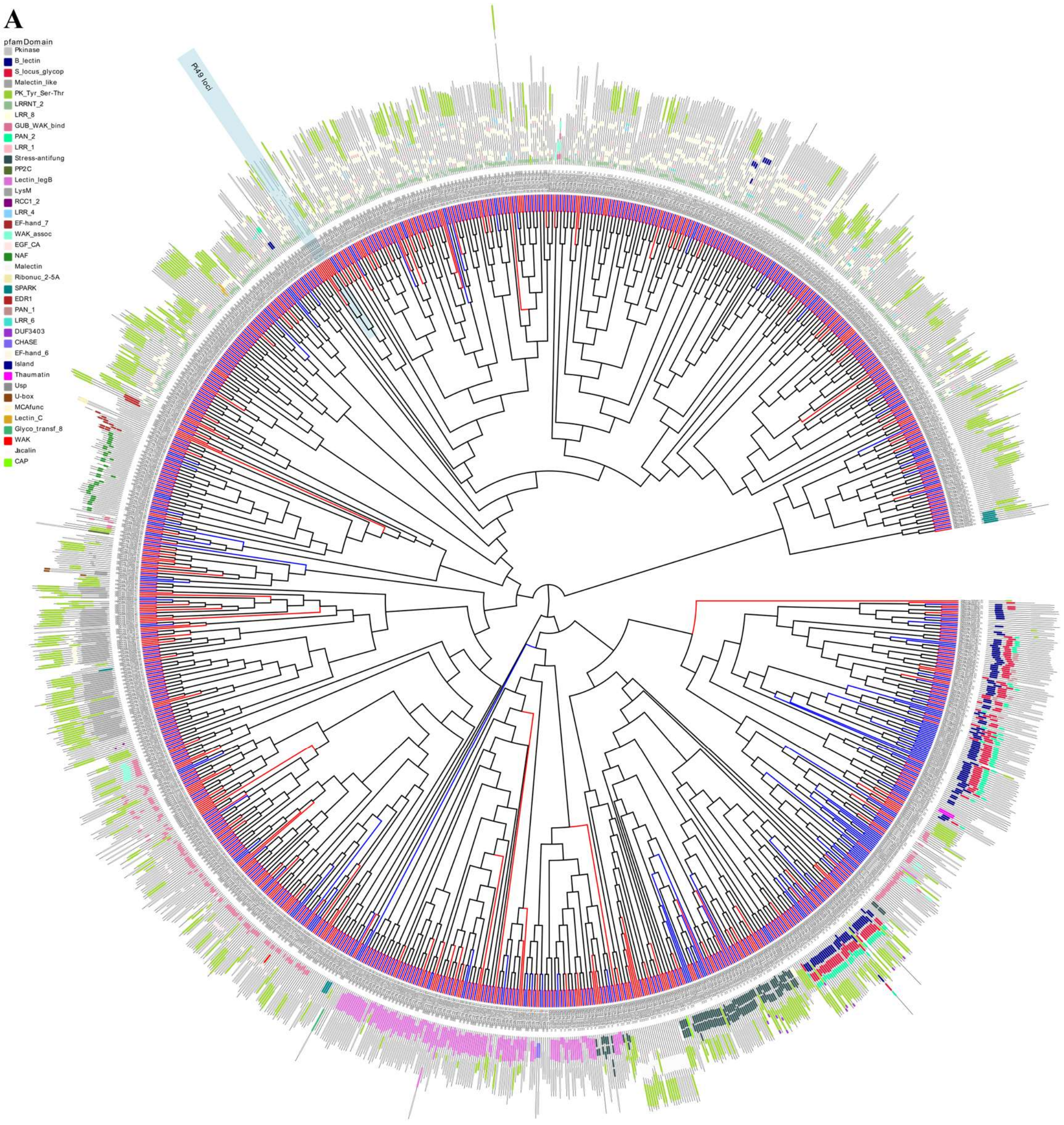
pfamDomain
Pkinase
B_lectin
S_locus_glycop
Malectin_like
PK_Tyr_Ser-Thr
LRRNT_2
LRR_8
GUB_WAK_bind
PAN_2
LRR_1
Stress-antifung
PP2C
Lectin_legB
LysM
RCC1_2
LRR_4
EF-hand_7
WAK_assoc
EGF_CA
NAF
Malectin
Ribonuc_2-5A
SPARK
EDR1
PAN_1
LRR_6
DUF3403
CHASE
EF-hand_6
Island
Thaumatin
Usp
U-box
MCAfunc
Lectin_C
Glyco_transf_8
WAK
Jacalin
CAP
Pi49 loci

B

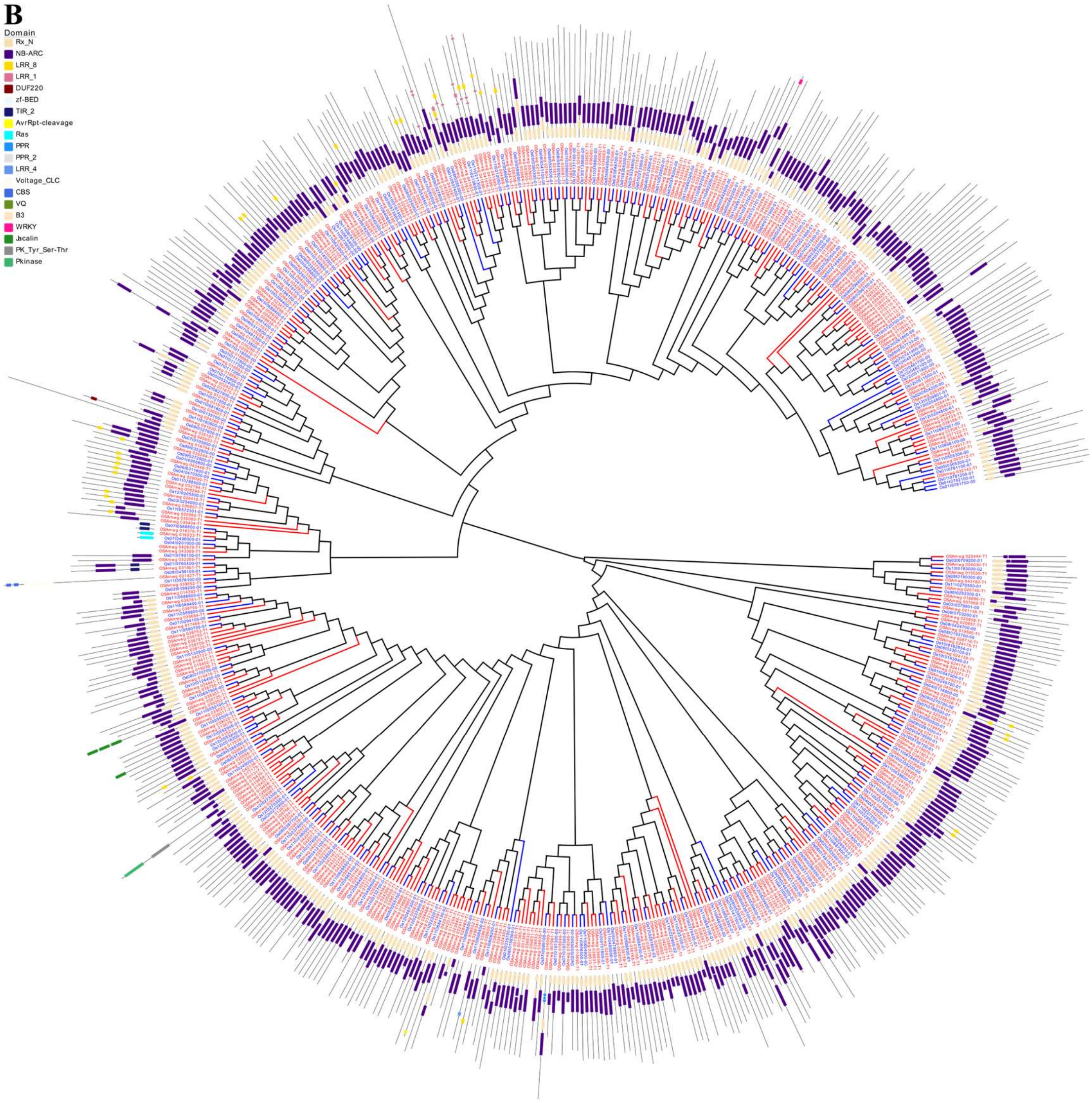
Domain
Rx_N
NB-ARC
LRR_8
LRR_1
DUF220
zf-BED
TIR_2
AvrRpt-cleavage
Ras
PPR
PPR_2
LRR_4
Voltage_CLC
CBS
VQ
B3
WRKY
Jacalin
PK_Tyr_Ser-Thr
Pkinase
