# Supplementary material for: The Genome of the Rice Variety Mowanggu Provides Insight Into Resistance to Magnaporthe oryzae
Source: Mol Plant Pathol. 2026 Mar 26;27(3):e70223. doi: 10.1111/mpp.70223 (PMC13097360; doi:10.1111/mpp.70223)
Supplement: Supplementary file 7 — Figure S7: Conserved sequence alignment of OSAmwg_038136 and orthologous genes between MWG and six rice cultivars. The grey‐shaded regions indicate identical amino acids among the different sequences, whereas the black‐shaded regions represent conserved replacements. [file MPP-27-e70223-s006.pdf]

```
                             *          20          *
MWG          : MKKPDFTASIDIADVISHRLVSYQEIIRATENFSIDNL :  38
IR64         : MKKPDFTASIDIADVISHRLVSYQEIIRATENFSNDNL :  38
IRGC26624    : MKKPDFTASIDIADVISHRLVSYQEIIRATENFSNDNL :  38
Zhonghua11   : MKKPDFTASIDIADVISHRLVSYQEIIRATENFSNDNL :  38
Shuhui498    : MKKPDFTASIDIADVISHRLVSYQEIIRATENFSNDNL :  38
9311         : MKKPDFTASIDIADVISHRLVSYQEIIRATENFSNDNL :  38
Tetep        : MKKPDFTASIDIADVISHRLVSYQEIIRATENFSIDNL :  38
               MKKPDFTASIDIADVISHRLVSYQEIIRATENFS DNL

               40          *          60          *
MWG          : LGVGSFGKVFKGRLDDGLCVAIKVLNMQVEQAIRTFDA :  76
IR64         : LGVGSFGKVFKGRLDDGLCVAIKVLNMQVEQAIRTFDA :  76
IRGC26624    : LGVGSFGKVFKGRLDDGLCVAIKVLNMQVEQAIRTFDA :  76
Zhonghua11   : LGVGSFGKVFKGRLDDGLCVAIKVLNMQVEQAIRTFDA :  76
Shuhui498    : LGVGSFGKVFKGRLDDGLCVAIKVLNMQVEQAIRTFDA :  76
9311         : LGVGSFGKVFKGRLDDGLCVAIKVLNMQVEQAIRTFDA :  76
Tetep        : LGVGSFGKVFKGRLDDGLCVAIKVLNMQVEQAIRTFDA :  76
               LGVGSFGKVFKGRLDDGLCVAIKVLNMQVEQAIRTFDA

                 80          *         100          *
MWG          : ECHVLRMARHRNLIKILNTCSNLDFRALLLQLMPNGSL : 114
IR64         : ECHVLRMARHRNLIKILNTCSNLDFRALLLQLMPNGSL : 114
IRGC26624    : ECHVLRMARHRNLIKILNTCSNLDFRALLLQLMPNGSL : 114
Zhonghua11   : ECHVLRMARHRNLIKILNTCSNLDFRALLLQLMPNGSL : 114
Shuhui498    : ECHVLRMARHRNLIKILNTCSNLDFRALLLQLMPNGSL : 114
9311         : ECHVLRMARHRNLIKILNTCSNLDFRALLLQLMPNGSL : 114
Tetep        : ECHVLRMARHRNLIKILNTCSNLDFRALLLQLMPNGSL : 114
               ECHVLRMARHRNLIKILNTCSNLDFRALLLQLMPNGSL
```
